# Associations of MALAT1 rs619586/rs3200401 and ANRIL rs10965215/rs10738605 polymorphisms with type 2 diabetes and diabetic nephropathy risk in an Egyptian population

**DOI:** 10.3389/fendo.2026.1802490

**Published:** 2026-06-12

**Authors:** Marwa A. Ali, Omayma O. Abdealeem, Olfat G. Shaker, Mahmoud G. Algammal, Nada F. Hemeda, Doaa Y. Ali, Mohammed Makloph, Mohamed M. Mohamed, Ahmed Magdy Elebiary, Asmaa Saleh, Marwa Kamal, Noura Mostafa Mohamed, Thanaa A. El-Masry

**Affiliations:** 1 Department of Biomedical Sciences, College of Medicine, King Faisal University, Al-Ahsa, Saudi Arabia; 2 Department of Medical Biochemistry and Molecular Biology, Faculty of Medicine, Fayoum University, Fayoum, Egypt; 3 Department of Medical Biochemistry and Molecular Biology, Faculty of Medicine, Cairo University, Cairo, Egypt; 4 Department of Internal Medicine, Faculty of Medicine, Fayoum University, Fayoum, Egypt; 5 Department of Genetics, Faculty of Agriculture, Fayoum University, Fayoum, Egypt; 6 Department of Clinical and Chemical Pathology, Faculty of Medicine, Fayoum University, Fayoum, Egypt; 7 Department of Forensic Medicine and Clinical Toxicology, Faculty of Medicine, Fayoum University, Fayoum, Egypt; 8 Department of Internal Medicine, Faculty of Medicine, Cairo University, Cairo, Egypt; 9 Department of Physiology, Faculty of Medicine, Fayoum University, Fayoum, Egypt; 10 Department of Pharmaceutical Sciences, College of Pharmacy, Princess Nourah bint Abdulrahman University, Riyadh, Saudi Arabia; 11 Department of Clinical Pharmacy, Faculty of Pharmacy, Fayoum University, Fayoum, Egypt; 12 Department of Basic Sciences, College of Medicine, Princess Nourah bint Abdulrahman University, Riyadh, Saudi Arabia; 13 Pharmacology and Toxicology Department, Faculty of Pharmacy, Tanta University, Tanta, Al-Gharbia, Egypt; 14 Pharmacology and Toxicology Department, Sinai University, Arish, Egypt

**Keywords:** diabetes mellitus, diabetic nephropathy, rs10738605, rs10965215, rs3200401, rs619586

## Abstract

**Background:**

Diabetic nephropathy (DN) is a serious microvascular complication of type 2 diabetes mellitus (T2DM).Single-nucleotide polymorphisms (SNPs) in long non-coding RNAs *MALAT1* and *ANRIL* may influence their expression and function, potentially affecting T2DM and DN risk.

**Objective:**

To investigate the association of *MALAT1* SNPs rs619586 and rs3200401 and *ANRIL* SNPs rs10965215 and rs10738605 with T2DM and/or DN susceptibility and clinical correlates in an Egyptian population.

**Methods:**

This case-control study included 64 T2DM patients, 75 DN patients, and 70 healthy controls from Fayoum University Hospital. Genomic DNA was extracted from peripheral blood, and SNP genotyping was performed using TaqMan real-time PCR. All analyses were adjusted for age and sex. The study had 80% power to detect associations based on expected differences in allele frequencies.

**Results:**

*MALAT1* rs619586 GG genotype and G allele were associated with reduced T2DM risk (adjusted odds ratio (AOR) 0.31, P = 0.004 and DN risk (AOR 0.32, P = 0.003). Conversely, *MALAT1* rs3200401 CT/TT genotypes increased T2DM risk (AOR 4.69, P<0.001) and DN risk (AOR 5.41, P<0.001). *ANRIL* rs10965215 GA/AA and rs10738605 CG genotypes were also associated with increased T2DM and DN risk versus controls. Notably, none of the SNPs were associated with DN progression among T2DM patients. This study is the first report of *ANRIL* SNPs in DN susceptibility.

**Conclusions:**

*MALAT1* rs619586 appears protective against T2DM and DN, while *MALAT1* rs3200401 and *ANRIL* rs10965215/rs10738605 increase susceptibility. These SNPs may serve as early risk markers pending validation in larger cohorts.

## 1 Introduction

Type 2 diabetes mellitus affects approximately 171 million people worldwide, and its prevalence is projected to rise rapidly in both developed and developing countries over the coming decades.

(1).Diabetic nephropathy (DN) is one of the most serious microvascular complications of diabetes mellitus, and it remains a major cause of morbidity and mortality worldwide (2). DN develops in approximately 20–40% of patients with diabetes (1). The global burden of DN continues to increase, with an estimated 107.6 million prevalent cases and 477, 300 deaths in 2021. The highest regional burdens have been reported in Southeast Asia (1, 739.3 per 100, 000), South Asia (1, 547.3 per 100, 000), and North Africa/Middle East (1, 505.9 per 100, 000) (3, 4).

Diabetic nephropathy (DN) is a leading cause of end-stage kidney disease worldwide. Although hyperglycemia, oxidative stress, inflammation, and hemodynamic disturbance are known contributors, the molecular mechanisms underlying DN remain incompletely understood (5). Long non-coding RNAs (lncRNAs) have emerged as important regulators of glucose metabolism, inflammation, and renal injury, making them attractive candidates for genetic and functional studies in diabetes and its complications (6).

Metastasis-associated lung adenocarcinoma transcript 1 (MALAT1) is among the best-studied lncRNAs in the context of diabetic complications. Experimental studies have shown that MALAT1 is dysregulated in diabetic nephropathy and contributes to podocyte injury, albuminuria, and renal fibrosis through pathways involving β-catenin, SRSF1, IL6ST, and Nox4/AMPK/mTOR signaling (7, 8). In addition, circulating and tissue MALAT1 expression has been reported to be altered in patients with diabetic kidney disease (9, 10)and other diabetic complications (11), supporting a broader pathogenic role for this transcript. Beyond expression changes, single-nucleotide polymorphisms in MALAT1 may also influence susceptibility to metabolic disease. In particular, rs619586 and rs3200401 have been associated with T2DM risk in Chinese Han populations, with rs619586 showing a protective effect and rs3200401 conferring increased risk (12).Also, MALAT1 SNP (rs619586) shows a protective effect against type 1 diabetes mellitus (13). Studies on the association between MALAT1 SNPs and DN are not available.

ANRIL, another lncRNA located at the 9p21 locus, has also been implicated in cardiometabolic disease (14) and diabetic complications (15). Experimental work suggests that ANRIL regulates a broad set of genes involved in extracellular matrix production, apoptosis, inflammatory signaling, and AGE-RAGE pathways, all of which are relevant to DN progression (15, 16). ANRIL knockout has been shown to attenuate albuminuria, polyuria, and multiple diabetes-associated renal abnormalities in animal models, indicating a direct role in diabetic kidney injury (16). However, data on ANRIL polymorphisms in DN susceptibility are limited, and the contribution of variants such as rs10965215 and rs10738605 has not been sufficiently explored.

Accordingly, this study investigated the association of MALAT1 rs619586 and rs3200401, and ANRIL rs10965215 and rs10738605, with susceptibility to T2DM and DN in an Egyptian population, and examined whether these variants correlate with clinical and laboratory measures relevant to renal involvement.

## 2 Patients and methods

### 2.1 Study design and subjects included

We designed our case-control study to involve three groups:T2DM, T2DM with nephropathy, and healthy individuals. 139 type 2 diabetic patients were diagnosed according to the American Standards of Care in Diabetes 2026 (17) and were recruited from outpatients of the Internal Medicine Department at Fayoum University Hospital, Al Fayoum, Egypt over the period from 6^th^ January 2023 to 30^th^July 2023.All practical experiments were done in the Medical Biochemistry, Microbiology, and Clinical Pathology departments at Fayoum University, Al Fayoum, Egypt. Individuals were categorized into groups based on specific health criteria to participate in this study.

Group 1 included patients with a confirmed diagnosis of Type 2 Diabetes Mellitus (T2DM). Inclusion criteria: age ≥18 years, T2DM duration ≥1 year, confirmed by medical history (age, sex, disease duration, treatments, smoking status, hypertension history), physical examination, and labs including Fasting Blood Glucose (FBG) ≥126 mg/dL, 2-hour postprandial glucose (2hpp) ≥200 mg/dL, cholesterol, triglycerides (TG), hemoglobin (HB), creatinine, urea, low-density lipoprotein (LDL), and high-density lipoprotein (HDL).Group 2 comprised patients with Type 2 Diabetes who also had confirmed Diabetic Nephropathy (DN). Inclusion criteria: T2DM per Group 1 plus KDIGO-confirmed DN (urine albumin-to-creatinine ratio (ACR) ≥30 mg/g or estimated glomerular filtration rate (eGFR) <60 mL/min/1.73 m² on ≥2 tests over 3 months) (18), diabetes duration ≥5 years. For both T2DM and DN groups, certain conditions led to exclusion from the study, including the presence of other microvascular or macrovascular diseases, any cancer, or autoimmune diseases.

Finally, the control group consisted of 70 healthy individuals. Inclusion criteria: age- and sex-matched to cases, age ≥18 years, normal FBG <100 mg/dL, normal ACR <30 mg/g, eGFR >90 mL/min/1.73 m², no acute/chronic diseases. Exclusion: any metabolic disorders, infections, or medications affecting glucose/kidney function.

### 2.2 Ethical approval

All participants provided written informed consent in accordance with the instructions of the Al Fayoum Ethics Committee, which granted the current study ethical approval number (R350), and the study was conducted in accordance with the Declaration of Helsinki.

### 2.3 DNA extraction and genotyping using real-time PCR

Five milliliters of venous blood were obtained from all individuals in EDTA-coated tubes. Genotyping was performed in subsequent steps. First, DNA was extracted using the QIAamp DNA extraction kit (Qiagen, Inc.) according to the manufacturer’s instructions. Second, thepurity and concentration of the extracted DNA were assessed using a NanoDrop-1000 Spectrophotometer (NanoDrop Technologies; Thermo Fisher Scientific, Inc.). Genotyping of 4 targeted SNPs using predesigned specific primers/probe sets from (Thermo Fisher Scientific, Inc.); (assay ID C_1060479_10(G/A)for MALAT1 rs619586 and C:_3246069_10 (T/C) for MALAT1 rs3200401), and assay ID: C:2618018_10 (A/G) for ANRIL rs10965215, and assay ID: C:11841819_10C/G for ANRIL rs10738605.

Genotyping of MALAT1 rs619586 and rs3200401 and ANRIL rs10965215 and rs10738605 was performed using predesigned TaqMan assays on a Rotor-Gene Q thermocycler.

Execution of four polymorphisms genotyping by Rotor Gene Q thermocycler (Qiagen) following these conditions: initial 15sec at 92˚C, then 40 PCR cycles; denaturation at 95˚C for 10 min; and annealing and extension at 60˚C for 90 sec.

### 2.4 Sample size calculation

The sample size was calculated *a priori* using OpenEpi 3.01 and Fleiss’ formula with continuity correction, based on 80% power, a two-sided alpha of 0.017, a 1:1 allocation ratio, and an expected 30% difference in allele frequency between cases and controls.

### 2.5 Statistical method

The collected data were statistically analyzed using SPSS software, version 27 (SPSS Inc., Chicago, IL, USA). Prior to statistical analysis, the distribution of continuous variables was assessed using the Shapiro–Wilk test. As laboratory data were collected only from the two independent case groups and not from a control group, comparisons were restricted to between-group analyses. Accordingly, most laboratory parameters were not normally distributed, comparisons between the two independent case groups were performed using the Mann–Whitney U test, and results were expressed as median and interquartile range (IQR). Hemoglobin (Hb), which followed an approximately normal distribution, was analyzed using the Student’s t-test and presented as mean ± standard deviation (SD).Within each case group, comparisons of laboratory parameters across different genotypes were performed using the Kruskal–Wallis test for non-normally distributed variables, while Hb was analyzed using one-way ANOVA where appropriate. *Post-hoc* pairwise comparisons were applied when overall significance was observed.

Hardy-Weinberg equilibrium (HWE) for the studied SNPs in the control group and Linkage disequilibrium (LD) for rs619586/rs3200401 and rs10965215/rs10738605 SNPs for the three study groups were estimated using Haploview 4.2 software. Haplotype blocks were identified using the Expectation-Maximization (EM) method.

For quantitative data, the mean and standard deviation (SD). An independent t-test or one-way ANOVA was used to test the significance of the results. Qualitative data were presented as numbers and percentages, and the chi-square (χ2) test was used for significance testing. Crude and adjusted Odds ratios (ORs) were calculated to assess the association between the study groups and genotype and allelic frequencies for the studied polymorphisms. Also, associations between the study groups and haplotype frequencies at the rs619586/rs3200401 and rs10965215/rs10738605 SNPs were assessed by calculating odds ratios (ORs) adjusted for age and sex, along with 95% confidence intervals (CIs). For the interpretation of significance test results, P < 0.05 was used as the cutoff.

### 2.6 Used genetic models

We used multiple genetic models to confirm the association between the target SNPs and the disease, whether T2DM or DN.

- Genotypic model:This model checks all three gene types separately: wild-wild (reference), wild-mutant, and mutant-mutant. It shows whether either of the changed types raises disease risk, without grouping them.
- Dominant model:Here, wild-wild is the reference. It groups wild-mutant and mutant-mutant together to test whether even a single mutant copy increases risk.
- Recessive model:People with at least one wild copy form the reference group. It checks only if two mutant copies (mutant-mutant) link to the disease.
- Allelic model:This approach skips gene pairs and directly compares single mutant versus wild gene copies across groups. It tests whether the mutant allele is associated with disease risk.

## 3 Results

The current study aims to compare the prevalence of target SNPs in the three studied groups (T2DM, DN, and control) to evaluate their association with diseases (T2DM and DN) and explore the association between these SNPs and clinicopathological characteristics of the disease.

### 3.1 Statistical analysis of demographic and laboratory data between the three studied groups (T2DM, DN, and controls)

The current study included 64 diabetic patients, 75 diabetic nephropathy patients, and 70 controls with matched age and sex (P>0.05). Statistical analysis of demographic and laboratory data between the DM and DN groups showed statistical significance regarding diabetes mellitus duration (median IQR = 9.5 (5.5-13.0) for DM, and 13 (8-28) for DN with P0.001). In addition, there was a significant increase in creatinine and urea serum levels in DN patients compared to DM patients (creatinine median IQR = 0.85 (0.69-1.02)for DM, 1.43 (1.08-2.3)for DN, P<0.001, and urea 28.5 (25-37.5)for DM, 39 (0.28-0.61) for DN, P<0.001). No other significance was detected (**Table 1**).

**Table 1:** Statistical demographic and laboratory data analysis between the three studied groups (T2DM, DN, and controls).

	Control		DM		Diabetic nephropathy		P-value
age	61.0	54-70	62.5	60-65.5	65	60-67	0.463
Sex							
Female	49	70.0%	40	62.5%	53	70.7%	0.538
Male	21	30.0%	24	37.5%	22	29.3%	
DM duration	-–	-–	9.5	5.5-13.0	13.0	8.0-28.0	0.001*
FBS			157.5	137.5-198.0	167.0	145-213.0	0.143
2HPP			279.0	240.5-363.5	300.0	254.0-376.0	0.163
Cholestero			167.0	122.5-204.5	160.0	120.0-196.0	0.666
TG			120.5	81.5-160.5	115.0	90.0-160.0	0.716
LDL			104.0	71.5-147.5	102.0	68.0-127.0	0.359
HDL			29.0	23.0-37.0	33.0	28.0-39.0	0.117
Creatinine			0.85	0.69-1.02	1.43	1.08-2.3	<0.001*
HB			12.7	1.7	12.7	1.6	0.966
urea			28.5	25-37.5	39	28.0-61.0	<0.001*

Data were expressed as number(%), median(intraquartile range) or mean ± SD.

T2DM, Type 2 diabetes mellitus; DN, Diabetic nephropathy; FBS, Fasting blood sugar; 2HPP, 2-hour postprandial; TG, triglycerides; LDL, low-density lipoprotein; HDL, High-density lipoprotein; HB, Hemoglobin. Note: Some data on controls are missing.

*P value <0.05. Bold values indicate statistical significance.

### 3.2 Statistical analysis of the prevalence of the two studied SNPs of MALAT1 (rs619586 and rs3200401), between the T2DM, DN, and controls

**Table 2** shows the prevalence of two MALAT1 SNPs (rs619586 and rs3200401) inT2DM, DN, and controls.

**Table 2:** MALAT1 SNPs: genotype frequencies and risk associations.

Variant		Control		T2DM		DN		T2DM vs. Control	DN vs Control	DN progression vs T2DM
								AOR (95% CI), p-value	AOR (95% CI), p-value	AOR (95% CI), p-value
		N	%	N	%	N	%			
rs619586**#** A>G	AA	12	17.1%	10	15.6%	16	21.3%	r	r	r
	AG	28	40.0%	42	65.6%	45	60.0%	1.959 (0.722, 5.312), 0.186	1.253 (0.514, 3.054), 0.620	0.648 (0.262, 1.598), 0.346
	GG	30	42.9%	12	18.8%	14	18.7%	0.528 (0.172, 1.621), 0.264	0.376 (0.139, 1.019), 0.054	0.704 (0.232, 2.141), 0.537
Dominant	AA	12	17.1%	10	15.6%	16	21.3%	r	r	r
	AG+GG	58	82.9%	54	84.4%	59	78.7%	1.276 (0.491, 3.318), 0.617	0.817 (0.352, 1.897), 0.639	0.660 (0.274 1.592, 0.355
Recessive	AA+AG	40	57.1%	52	81.3%	61	81.3%	r	r	r
	GG	30	42.9%	12	18.8%	14	18.7%	0.311 (0.141, 0.694), 0.004*	0.319 (0.150, 0.681), 0.003*	0.986 (0.417, 2.329), 0.974
Allele	A	52	37.1%	62	48.4%	77	51.3%	r	r	r
	G	88	62.0%	66	51.6%	73	48.7%	0.656 (0.399, 1.078), 0.096	0.585 (0.364, 0.940), 0.027*	0.879 (0.547, 1.414), 0.596
rs3200401**##** C>T	CC	36	51.4%	12	18.8%	12	16.0%	r	r	r
	CT	20	28.6%	32	50.0%	27	36.0%	4.900 (2.050, 11.715), <0.001*	3.926 (1.634, 9.433), 0.002*	0.814 (0.312, 2.123), 0.675
	TT	14	20.0%	20	31.3%	36	48.0%	4.403 (1.698, 11.416), 0.002*	7.536 (3.059, 18.563), <0.001*	1.688 (0.634, 4.492), 0.294
Dominant	CC	36	51.4%	12	18.8%	12	16.0%	r	r	r
	CT+TT	34	48.6%	52	81.3%	63	84.0%	4.693 (2.214, 10.369), <0.001*	5.414 (2.487, 11.788), <0.001*	1.149 (0.472, 2.797), 0.759
Recessive	CC+CT	56	80.0%	44	68.8%	39	52.0%	r	r	r
	TT	14	20.0%	20	31.3%	36	48.0%	1.853 (0.838, 4.099), 0.128	3, 665 (1.742, 7.711), <0.001*	1.953 (0.965, 3.951), 0.063
Allele	C	92	65.7%	56	43.8%	51	34.0%	**r**	**r**	r
	T	48	34.3%	72	56.3%	99	66.0%	2.492 (1.516, 4.095), <0.001*	3.661 (2.248, 5.964), <0.001*	1.452 (0.889, 2.371), 0.136

**#**HWE p-value for control: 0.313, **##**HWE p-value for control: 0.004*. T2DM, Type 2 Diabetes Mellitus; DN, Diabetic Nephropathy, AOR, adjusted odds ratio; CI, Confidence Interval.*P value <0.05. Significant values were indicated as bold.

For the rs619586 SNP, results showed that the homozygous mutant GG genotype or the G allele was associated with a lower risk of T2DM and DN compared with controls. This association is obvious in recessive and allelic models. In the recessive model, GG carriers (versus AA+AG reference) had adjusted odds ratios (AORs) of 0.311 (95% CI: 0.141-0.694) for T2DM and 0.319 (95% CI: 0.150-0.681) for DN. In the allelic model, the G allele (versus A reference) showed an AOR of 0.585 (95% CI: 0.364-0.940) for DN. These findings indicate the rs619586 variant may protect against T2DM and/or DN.

For rs3200401, the genotypic model showed that carrying the heterozygous CT or homozygous mutant TT genotype significantly increased the risk of both T2DM and DN compared with the CC reference genotype among controls. The TT genotype posed a higher risk than CT, with adjusted odds ratios (AORs) of 7.536 (95% CI: 3.059-18.563, p<0.001) for TT, versus 3.926 (95% CI: 1.634-9.433, p=0.002) for CT in DN.

This risk pattern held across other models. In the dominant model, mutant carriers (CT+TT vs. CC) had AORs of 4.693 (95% CI: 2.214-10.369, p<0.001) for T2DM and 5.414 (95% CI: 2.487-11.788, p<0.001) for DN. The allelic model (T vs. C allele) showed AORs of 2.492 (95% CI: 1.516-4.095, p<0.001) for T2DM and 3.661 (95% CI: 2.248-5.964, p<0.001) for DN. The recessive model indicated a 3.665-fold increased DN risk for TT carriers (95% CI: 1.742-7.711, p<0.001).

These findings suggest that thevariant rs3200401 significantly increases susceptibility to T2DM and/or DN in healthy individuals. The risk is associated with both heterozygous and homozygous mutant genotypes, with the latter carrying a higher risk in most cases.

MALAT1 rs619586 was associated with reduced risk of T2DM (AOR 0.31, 95% CI 0.14-0.69) and DN versus controls (AOR 0.32, 95% CI 0.15-0.68), while rs3200401 increased both risks (dominant model AOR 4.69 for T2DM, 5.41 for DN).

### 3.3 Statistical analysis of the prevalence of the two studied SNPs of ANRIL (rs10965215 and rs10738605) between the T2DM, DN, and controls

**Table 3** shows the distribution of ANRILSNPs (rs10965215 and rs10738605) betweenT2DM, DN, and controls.

**Table 3:** ANRIL SNPs: genotype frequencies and risk associations.

Variant		Control		T2DM		DN		T2DM vs. Control	DN vs Control	DN progression vs T2DM
								AOR (95% CI), p-value	AOR (95% CI), p-value	AOR (95% CI), p-value
		N	%	N	%	N	%			
rs10965215# G>A	GG	22	31.4%	10	15.6%	10	13.3%	r	r	r
	GA	20	28.6%	32	50.0%	40	53.3%	3.597 (1.405, 9.205), 0.008*	4.552 (1.795, 11.546), 0.001*	1.220 (0.449, 3.317), 0.697
	AA	28	40.0%	22	34.4%	25	33.3%	1.782 (0.696, 4.562), 0.30	1.917 (0.757, 4.854), 0.170	1.053 (0.365, 3.037), 0.924
Dominant	GG	22	31.4%	10	15.6%	10	13.3%	r	r	r
	GA+AA	48	68.6%	54	84.4%	65	86.7%	2.543 (1.088, 5.947), 0.031*	2.987 (1.289, 6.919), 0.011*	1.152 (0.443, 2.996), 0.772
Recessive	GG+GA	42	60.0%	42	65.6%	50	66.7%	r	r	r
	AA	28	40.0%	22	34.4%	25	33.3%	0.797 (0.393, 1.617), 0.530	0.723 (0.364, 1.437), 0.355	0.901 (0.440, 1.845), 0.776
Allele	G	64	45.7%	52	40.6%	60	40.0%	r	r	r
	A	76	54.3%	76	59.4%	90	60.0%	1.251 (0.768, 2.037), 0.368	1.245 (0.779, 1.991), 0.359	0.989 (0.609, 1.606), 0.963
rs10738605## C>G	CC	30	42.9%	14	21.9%	14	18.7%	r	r	r
	CG	20	28.6%	44	68.8%	55	73.3%	5.287 (2.243, 12.461), <0.001*	5.954 (2.615, 13.554), <0.001*	1.183 (0.507, 2.763), 0.698
	GG	20	28.6%	6	9.4%	6	8.0%	0.721 (0.232, 2.243), 0.572	0.635 (0.208, 1.943), 0.426	0.930 (0.238, 3.720), 0.930
Dominant	CC	30	42.9%	14	21.9%	14	18.7%	r	r	r
	CG+GG	40	57.1%	50	78.1%	61	81.3%	3.027 (1.375, 6.666), 0.006*	3.273 (1.540, 6.959), 0.002*	1.155 (0.499, 2.671), 0.736
Recessive	CC+CG	50	71.4%	58	90.6%	69	92.0%	r	r	r
	GG	20	28.6%	6	9.4%	6	8.0%	0.264 (0.098, 0.714), 0.009*	0.214 (0.080, 0.574), 0.002*	0.824 (0.247, 2.749), 0.753
Allele	C	80	57.1%	72	56.3%	83	55.3%	r	r	r
	G	60	42.9%	56	43.8%	67	44.7%	1.082 (0.663, 1.767), 0.751	1.071 (0.671, 1.709), 0.773	1.017 (0.629, 1.642), 0.946

#HWE p-value for control: 0.0006*, ##HWE p-value for control: 0.0008*.T2DM, Type 2 Diabetes Mellitus; DN, Diabetic Nephropathy, AOR, adjusted odds ratio; CI, Confidence Interval. *P value <0.05. Bold values indicate statistical significance.

The rs10965215 variant was associated with increased risk of T2DM and/or DN among controls. Compared to the wild-type GG genotype, carrying the heterozygous AG or homozygous mutant AA genotype significantly raised this risk, with the pattern consistent across genotypic and dominant models.Adjusted odds ratios (AORs) for T2DM were 3.597 (genotypic model) and 2.543 (dominant model, AG+AA vs. GG). For DN, they were 4.552 (genotypic) and 2.543 (dominant). All p-values were statistically significant (<0.05).

For the rs10738605 variant, the genotypic model showed that the heterozygous CG genotype carried nearly a 5-fold higher risk of T2DM (AOR: 5.287, 95% CI: 2.243-12.461, p<0.001) and a 6-fold higher risk of DN (AOR: 5.954, 95% CI: 2.615-13.554, p<0.001) compared to the wild-type CC genotype in controls.The dominant model (CG+GG vs. CC) confirmed almost 3-fold increased risks, with AORs of 3.027 (95% CI: 1.375-6.666, p=0.006) for T2DM and 3.273 (95% CI: 1.540-6.959, p=0.002) for DN.Unexpectedly, the recessive model indicated that CG carriers had a reduced risk of T2DM (AOR: 0.264, 95% CI: 0.098-0.714, p=0.009) and DN (AOR: 0.214, 95% CI: 0.080-0.574, p=0.002).

Overall, rs10965215 and rs10738605 are generally associated with an increased risk of T2DM and DN. However, unexpectedly, CG shows a reduced risk in the recessive model, requiring further investigation. To summarize, neither ANRIL rs10965215 nor rs10738605 was associated with DN progression among T2DM patients (all genetic models vs. T2DM group: AOR 0.90-1.22, p>0.35), consistent with a role in disease susceptibility rather than progression to nephropathy.

All four SNPs showed significant associations with T2DM and DN risk when comparing cases to healthy controls. However, when comparing DN patients directly to T2DM patients without nephropathy, none of the SNPs were associated with DN progression (**Tables 2**, **3**: all DN vs. T2DM comparisons p>0.05). This null finding suggests that MALAT1 and ANRIL variants primarily influence susceptibility to T2DM/DN rather than progression from diabetes to nephropathy among diabetic patients.

### 3.4 Haplotypes analysis

Haplotype analysis of rs619586 and rs3200401 polymorphisms compared T2DM or DN patients to controls (or T2DM to DN patients), using the wild-type GC haplotype as reference (**Table 4**), and revealed key risk associations.

**Table 4:** Haplotype analysis of MALAT1 SNPs (rs619586A/G) and (rs3200401C/T) in T2DM and DN compared to healthy controls.

Haplotype	T2DM, control	P-value	AOR	95% CIfor AOR	
**GC**	0.217, 0.415	r			
**GT**	0.299, 0.214	0.004*	**2.624**	**1.359**	**5.065**
**AC**	0.221, 0.243	0.120	1.706	0.87	3.345
**AT**	0.264, 0.129	<0.001*	**3.913**	**1.89**	**8.102**
**Constant**		0.002	0.483		
	DN, control	P-value	AOR	95% CIfor AOR	
**GC**	0.159, 0.415	r			
**GT**	0.327, 0.213	<0.001*	**3.947**	**2.045**	**7.62**
**AC**	0.333, 0.130	<0.001*	**6.713**	**3.272**	**13.773**
**AT**	0.181, 0.242	0.066	1.919	0.959	3.841
**Constant**		<0.001	0.414		
	DN, T2DM	P-value	AOR	95% CIfor AOR	
**GT**	0.335, 0.313	r			
**AT**	0.325, 0.250	0.514	1.225	0.666	2.253
**AC**	0.189, 0.235	0.387	0.747	0.385	1.447
**GC**	0.151, 0.203	0.332	0.708	0.352	1.423
**Constant**		0.293	1.25		

T2DM, Type 2 Diabetes Mellitus; DN, Diabetic Nephropathy, AOR, adjusted odds ratio; CI, Confidence Interval. *P value <0.05. Bold values indicate statistical significance.

For T2DM risk versus controls, the GT haplotype (rs619586 G + rs3200401 T) showed an AOR of 2.624 (95% CI: 1.359-5.065, p=0.004), while the AT haplotype (rs619586 A + rs3200401 T) had an AOR of 3.913 (95% CI: 1.89-8.102, p<0.001); however, other haplotypes like GC itself or certain combinations showed no significant association with T2DM risk.

Similarly, for DN risk versus controls, the GT haplotype (rs619586 G + rs3200401 T) carried an AOR of 3.947 (95% CI: 2.045-7.62, p<0.001), and the AC haplotype (rs619586 A + rs3200401 C) showed an AOR of 6.713 (95% CI: 3.272-13.773, p<0.001), whereas remaining haplotypes lacked significant links to DN.

In conclusion, this haplotype analysis identifies GT, AT, and AC as risk factors for T2DM or DN susceptibility relative to GC. In contrast, other haplotypes show no association, suggesting that these combinations at rs619586 and rs3200401 are potential biomarkers warranting validation in larger, diverse cohorts.

**Figure 1:**
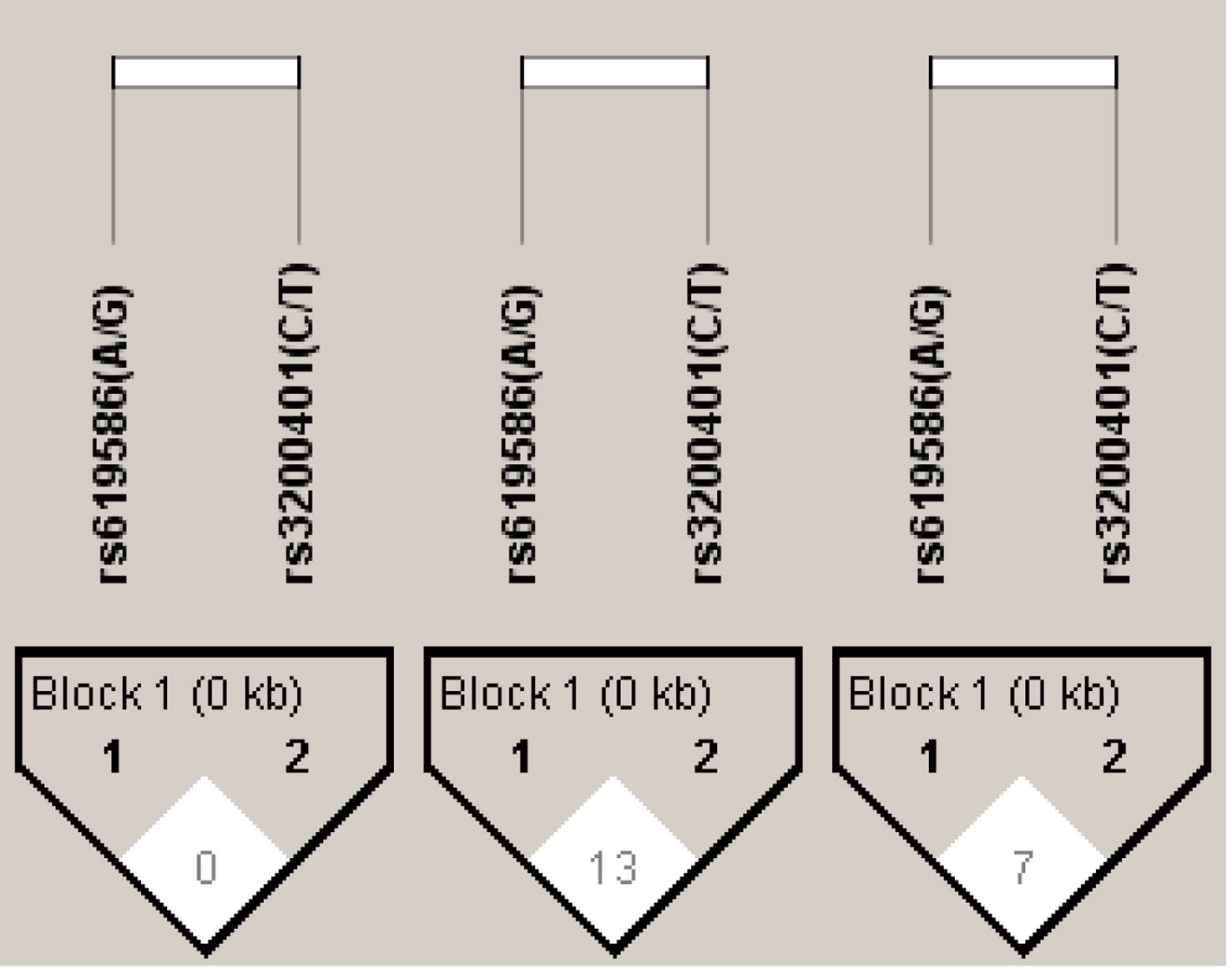
Linkage disequilibrium (LD) between MALAT1 SNPs rs619586 and rs3200401 Panel **(A)** Controls (D’ = 0.125, 95% CI 0.01-0.32) Panel **(B)** T2DM patients (D’ = 0.139, 95% CI 0.01-0.39) Panel **(C)** DN patients (D’ = 0.079, 95% CI 0.00-0.33) LD plots showing recombination patterns between MALAT1 rs619586 (A>G) and rs3200401 (C>T) across study groups. Low D’ values (<0.15) indicate weak LD, suggesting these SNPs are largely inherited independently. The slightly higher LD in T2DM (Panel **(B)**) compared with controls may reflect population stratification or selection pressures.

**Figure 1** shows linkage disequilibrium (LD) between the two MALAT1 SNPS for controls, T2DM and DN, respectively: LD between rs619586 and rs3200401, in control: D’ = 0.125 (95% CI: 0.01-0.32), in T2DM: D’ = 0. 0.139 (95% CI: 0.01-0.39), in DN: D’ = 0.079 (95% CI: 0.0-0.33).

Low D’ values (<0.2) across all groups indicate weak LD between these SNPs, meaning they are inherited independently rather than as a block. These findings justify separate haplotype analysis, as the variants do not strongly tag each other.

Haplotype analysis of rs10965215 and rs10738605 polymorphisms compared T2DM or DN patients to controls (or T2DM to DN patients), using the wild-type GC haplotype as reference (**Table 5**), and showed that the GG haplotype (rs10965215 G allele + rs10738605 G allele) significantly reduced DN risk among controls, with an odds ratio (OR) of 0.315 (95% CI: 0.131-0.753, p=0.009).

**Table 5:** Haplotype analysis of ANRIL SNPs (rs10965215G/A) and (rs10738605C/G) in T2DM and DN compared to healthy controls.

Haplotype	T2DM, control	P-value	AOR	95% CIfor AOR	
**GC**	0.292, 0.289	r			
**AG**	0.323, 0.260	0.519	1.231	0.654	2.318
**AC**	0.271, 0.283	0.864	0.946	0.50	1.788
**GG**	0.115, 0.169	0.328	0.676	0.308	1.481
**Constant**		0.733	0.925		
	DN, control	P-value	AOR	95% CIfor AOR	
**GC**	0.340, 0.293	r			
**AG**	0.386, 0.264	0.436	1.260	0.704	2.256
**AC**	0.214, 0.279	0.190	0.660	0.354	1.229
**GG**	0.060, 0.165	0.009*	**0.315**	**0.131**	**0.753**
**Constant**		0.298	0.315		
	DN, T2DM	P-value	AOR	95% CIfor AOR	
**AG**	0.420, 0.372	r			
**GC**	0.373, 0.341	0.912	0.970	0.562	1.672
**AC**	0.180, 0.222	0.351	0.735	0.384	1.405
**GG**	0.027, 0.066	0.133	0.381	0.108	1.340
**Constant**		0.156	1.312		

T2DM, Type 2 Diabetes Mellitus; DN, Diabetic Nephropathy, AOR, adjusted odds ratio; CI, Confidence Interval. *P value <0.05. Bold values indicate statistical significance.

In conclusion, the protective GG haplotype versus GC highlights rs10965215-rs10738605 combinations as potential factors that lower DN susceptibility, warranting further research for risk stratification in diabetic populations.

**Figure 2:**
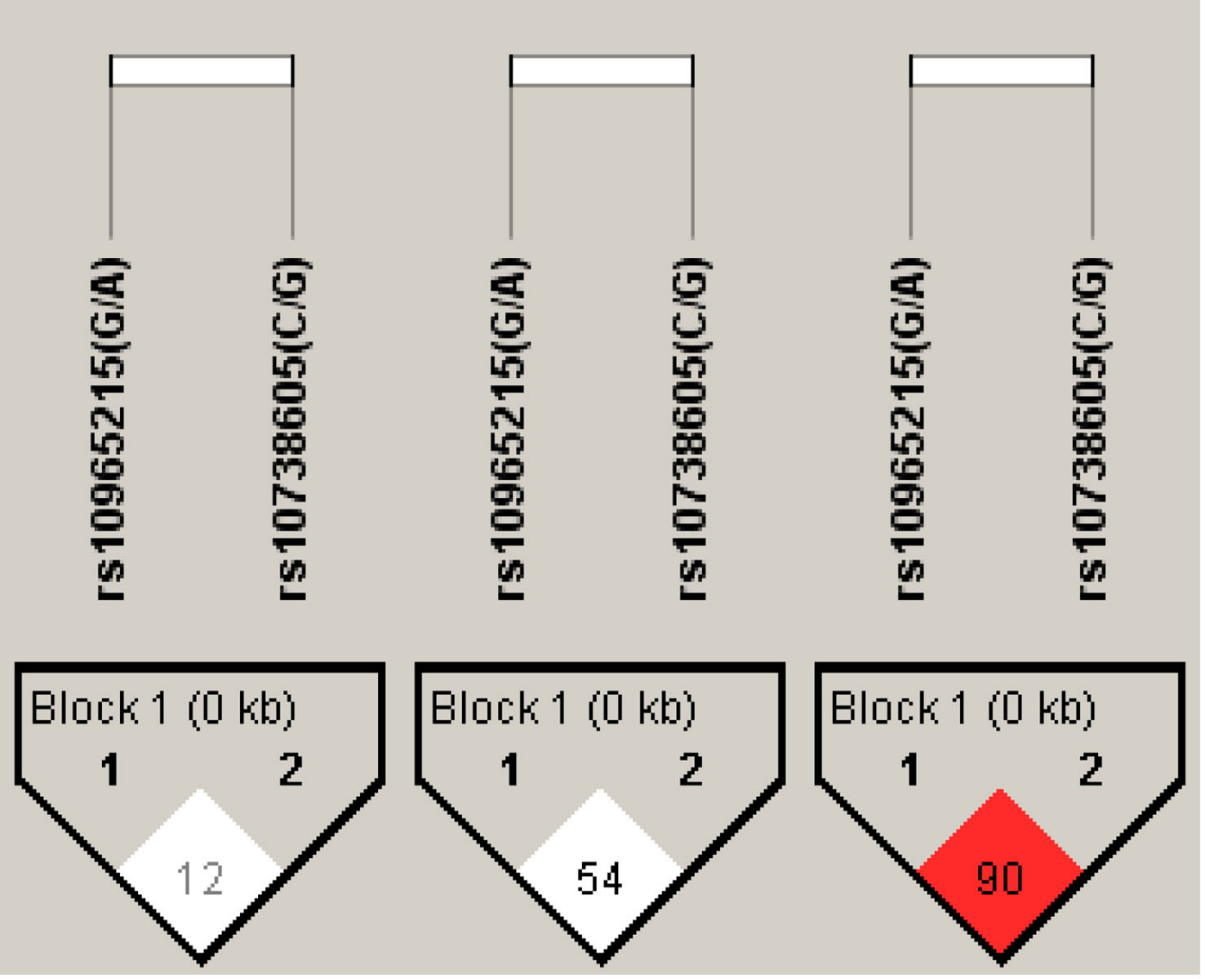
Linkage disequilibrium (LD) between ANRIL SNPs rs10965215 and rs10738605 Panel **(A)** Controls (D’ = 0.125, 95% CI 0.01-0.32) Panel **(B)** T2DM patients (D’ = 0.54, 95% CI 0.20-0.74) Panel **(C)** DN patients (D’ = 0.903, 95% CI 0.72-0.97) LD plots demonstrating progressively stronger linkage between ANRIL rs10965215 (G>A) and rs10738605 (C>G) from controls to disease groups. Moderate LD in T2DM (D’ = 0.54) escalates to strong LD in DN (D’ = 0.90), suggesting disease-associated haplotypes or population substructure.

**Figure 2** shows linkage disequilibrium (LD) of ANRIL two SNPs for controls, T2DM and DN, respectively: Linkage disequilibrium (LD) between rs10965215and rs10738605, in control: D’ = 0.125 (95% CI: 0.01-0.32), in T2DM: D’ = 0.54 (95% CI: 0.2-0.74), In DN: D’ = 0.903 (95% CI: 0.72-0.97).These D’ values indicate weak LD in controls (low co-inheritance), moderate LD in T2DM, and strong LD in DN (near-complete linkage).

### 3.5 The association between the 4 studied SNPs and clinical characteristics of T2DM and DN groups.

**Tables 6**–**9** represent statistical analysis of the association between the 4studied SNPs and clinicopathological characters in the T2DM group and DN group.

**Table 6:** Association between MALAT1 SNPS and characteristics in T2DM patients.

Parameter	rs619586						P-value	Allele				P-value
	AA		AG		GG			A		G		
DM duration	10.1	6	9.9	5.3	10.6	7.7	0.940	9.97	5.43	10.15	6.12	0.858
FBS	153.4	58.6	165.3	44.9	172.8	54.8	0.653	161.47	49.01	168.02	48.01	0.447
2HPP	300.1	114.3	296	87.3	302.3	93.9	0.975	297.34	94.76	298.32	88.31	0.952
Cholesterol	182.9	65.5	158.1	54.8	194.8	69.9	0.131	166.13	58.5	171.45	62.17	0.618
TG	146.6	59.8	126.4	58.5	128.9	37.9	0.568	132.89	58.69	127.29	51.43	0.566
LDL	123.6	55.2	102.1	50.7	142.8	70.8	0.073	109.01	52.26	116.88	60.88	0.436
HDL	30.2	8.5	30.5	9.4	26.9	12.5	0.538	30.42	8.98	29.21	10.58	0.489
Creatinine	0.9	0.1	0.9	0.3	0.9	0.4	0.813	0.85	0.22	0.87	0.32	0.668
HB	13.8	1.9	12.6	1.6	12	1.3	0.039*	12.96	1.78	12.38	1.51	0.049*
urea	32.4	7.6	29.5	9.1	32.3	10.1	0.495	30.43	8.62	30.49	9.4	0.971
Parameter	rs3200401						P-value	Allele				P-value
	CC		CT		TT			C		T		
DM duration	12.3	8	9.4	4.5	9.8	6.1	0.321	10.66	6.27	9.6	5.36	0.303
FBS	174.6	50.5	163.3	47.4	161.4	51.1	0.742	168.16	48.15	162.26	48.8	0.496
2HPP	337.9	101.2	288.8	76.3	288.3	105.3	0.246	309.82	89.34	288.53	92.03	0.191
Cholesterol	190.1	68	161.8	51.8	167.4	68.6	0.388	173.93	59.65	164.94	60.82	0.404
TG	144.8	52.9	117.4	50.7	141.3	61	0.185	129.14	52.51	130.67	57.07	0.877
LDL	134.6	65.9	104.6	46	113.6	66.3	0.303	117.5	56.15	109.63	57.42	0.439
HDL	28.3	11.5	32.6	9.1	26.2	9	0.062	30.76	10.24	29.04	9.48	0.326
Creatinine	0.8	0.2	0.9	0.3	0.9	0.3	0.676	0.86	0.24	0.87	0.3	0.822
HB	12.6	1.4	12.6	2	12.8	1.2	0.875	12.59	1.76	12.72	1.61	0.648
urea	30.1	7.1	30.9	9.2	30	10.2	0.926	30.54	8.23	30.39	9.6	0.925

FBS, Fasting blood sugar; 2HPP, 2-hour postprandial; TG, triglycerides; LDL, low-density lipoprotein; HDL, High-density lipoprotein; HB, Hemoglobin. Note: Some data on controls are missing. Bold values indicate statistical significance.

**Table 7:** Association between MALAT1 SNPS and characteristics in DN patients.

Parameter	rs619586						P-value	Allele				P-value
	AA		AG		GG			A		G		
DM duration	14.2	9.7	19.7	15.7	17.9	15.8	0.435	17.42	13.67	19	15.54	0.508
FBS	183.1	57.7	175.6	46.3	177.8	47.5	0.873	178.71	50.69	176.45	46.14	0.776
2HPP	310.4	83.2	324.7	86.1	305.1	63.5	0.677	318.75	84.1	317.15	77.95	0.904
Cholesterol	180.3	54.4	156.3	51.8	162.6	51.9	0.295	166.29	53.47	158.71	51.18	0.377
TG	158.1	83.8	127.4	57.9	152.9	74.9	0.208	140.17	70.29	137.22	65.02	0.790
LDL	115.4	54.8	98	45.4	100	50.3	0.460	105.21	49.52	98.73	46.63	0.411
HDL	33	13.1	31.6	9.6	31.6	9.3	0.898	32.21	11.01	31.64	9.38	0.737
Creatinine	1.9	1.2	2.1	1.9	1.9	1.2	0.854	2.03	1.6	2.01	1.63	0.949
HB	12.4	1.8	12.7	1.3	12.7	2.3	0.845	12.6	1.5	12.7	1.71	0.715
urea	50.3	30	49.1	34.4	52.7	40.5	0.942	49.6	32.26	50.5	36.31	0.872
Parameter	rs3200401						P-value	allele				P-value
	CC		CT		TT			C		T		
DM duration	17.3	13	15.6	13.4	20.5	16	0.412	16.35	12.96	19.13	15.33	0.270
FBS	197.8	57.9	184.1	46.2	166	45.1	0.099	190.55	51.29	170.95	45.65	0.018*
2HPP	**373.3**	**77.9**	309.6	71.2	305.8	83.7	0.034*	339.57	79.61	306.85	79.68	0.019*
Cholesterol	150	52.4	165.4	62.1	164.7	45.1	0.686	158.18	57.22	164.88	49.78	0.459
TG	133.8	80.1	150.3	73.9	131.7	59	0.543	142.53	75.69	136.78	63.29	0.623
LDL	90.6	45.1	103.4	59.7	104.9	39.7	0.669	97.38	52.86	104.46	45.53	0.395
HDL	32.6	13.9	30.9	10.7	32.5	8.7	0.795	31.67	12.05	32.07	9.21	0.834
Creatinine	1.5	0.9	2.3	2.3	2	1.1	0.403	1.93	1.79	2.06	1.52	0.632
HB	13.2	1.4	12.4	1.6	12.7	1.7	0.294	12.78	1.53	12.58	1.64	0.485
urea	49.2	30.4	43.6	28.4	55.2	39.2	0.419	46.24	28.85	51.99	36.61	0.330

FBS, Fasting blood sugar; 2HPP, 2-hour postprandial; data on controls are missing. Bold values indicate statistical significance.

**Table 8:** Association between ANRIL SNPS and characteristics in T2DM patients.

Parameter	rs10965215						P-value	Allele				P-value
	GG		GA		AA			G		A		
DM duration	10.8	6.4	10.1	6.2	9.7	5.1	0.833	10.37	6.15	9.86	5.54	0.626
FBS	189.3	42.4	150.1	47.2	175.1	48	0.050	165.19	48.55	164.61	48.65	0.947
2HPP	353	80	269.8	95.7	313.5	77.1	0.024*	301.83	97.46	295.12	87.09	0.684
Cholesterol	131.2	51.1	177.9	52.2	172.8	70.8	0.094	159.96	55.74	174.97	62.76	0.167
TG	113.1	57.7	129.9	50.9	137.9	60.5	0.507	123.42	53.09	134.5	56.03	0.264
LDL	77.8	39.4	122.5	52.2	115.3	65.8	0.092	105.33	51.83	118.36	59.69	0.204
HDL	27.5	8.4	29.4	11.2	31.4	8.4	0.565	28.68	10.11	30.56	9.61	0.289
Creatinine	0.9	0.2	0.9	0.3	0.8	0.2	0.906	0.87	0.28	0.86	0.27	0.719
HB	11.9	1.2	12.4	1.8	13.3	1.5	0.051	12.24	1.59	12.95	1.67	0.018*
urea	31.1	10.9	31.7	9.5	28.4	7.2	0.423	31.44	9.86	29.79	8.35	0.311
Parameter	rs10738605						P-value	Allele				P-value
	CC		CG		GG			C		G		
DM duration	9.4	5.7	10.4	5.7	9.5	7.5	0.831	9.97	5.66	10.18	5.98	0.842
FBS	154.8	39.2	161.1	49.4	216	34.9	0.021*	158.62	45.29	172.84	51.47	0.100
2HPP	289.8	78.2	292	96.9	359.2	61.7	0.228	291.17	89.04	306.43	93.85	0.349
Cholesterol	175.8	77	159.9	52.6	218.8	54.8	0.070	166.06	62.54	172.5	57.5	0.550
TG	130.2	58.4	124.9	54.6	167.2	43.7	0.214	126.94	55.34	133.93	54.62	0.478
LDL	122.6	71.7	105	50.3	150.2	57.8	0.148	111.83	59.08	114.66	54.17	0.781
HDL	29.4	10.1	29.2	10.3	35.3	3.4	0.357	29.25	10.06	30.5	9.55	0.477
Creatinine	0.9	0.3	0.8	0.3	1	0.2	0.516	0.86	0.29	0.87	0.26	0.782
HB	12.2	0.5	12.7	1.9	13.7	1.5	0.184	12.48	1.52	12.89	1.83	0.173
urea	28.7	8.8	31	9.6	30.2	4.9	0.710	30.15	9.23	30.86	8.74	0.659

FBS, Fasting blood sugar; 2HPP, 2-hour postprandial; TG, triglycerides; LDL, low-density lipoprotein; HDL, High-density lipoprotein; HB, Hemoglobin. Note: Some data on controls are missing. Bold values indicate statistical significance.

**Table 9:** Association between ANRIL SNPS and characteristics in DN patients.

Parameter	rs10965215						P-value	Allele				P-value
	GG		GA		AA			G		A		
DM duration	20.3	14.1	16.6	13.5	19.8	16.7	0.627	17.87	13.59	18.4	15.27	0.827
FBS	175.7	45.1	175.7	47.1	181.4	53.6	0.893	175.7	45.7	178.89	50.3	0.694
2HPP	332.5	96.5	310.4	77	324.2	83.4	0.671	317.78	82.91	318.1	80	0.981
Cholesterol	166.4	45.1	156.3	51.2	171.1	57.7	0.533	159.68	48.78	164.54	54.75	0.579
TG	123.4	54.2	136.4	66.1	148.6	75.9	0.587	132.07	61.81	143.18	71.13	0.325
LDL	107.1	34.2	96.4	47.4	109.1	54.5	0.558	99.97	43.24	103.45	51.25	0.666
HDL	34.6	9.9	31	10.9	32.4	9.5	0.590	32.18	10.55	31.77	10.05	0.808
Creatinine	2.3	1.1	1.9	1.6	2	1.8	0.802	2.06	1.48	1.99	1.7	0.784
HB	13.1	1.1	12.5	1.9	12.6	1.3	0.652	12.73	1.64	12.6	1.58	0.637
urea	66.1	40.3	44.9	30	51.8	37.4	0.210	52	34.5	48.73	34.09	0.568
Parameter	rs10738605						P-value	Allele				P-value
	CC		CG		GG			C		G		
DM duration	19.2	16.1	18.9	14.6	8.8	8.6	0.266	19.04	14.92	17.13	14.19	0.429
FBS	189.4	58	176.4	47	161	39	0.463	180.81	50.6	173.66	45.55	0.370
2HPP	342.7	92.2	312.8	80.7	307.8	54.9	0.451	322.88	84.75	311.9	76.06	0.410
Cholesterol	159.4	42.4	164	51.3	157.3	87.2	0.930	162.45	48.04	162.79	57.57	0.968
TG	130.7	67.4	145	69.2	99.8	45.9	0.270	140.19	68.09	136.93	67.37	0.770
LDL	99.3	35.1	102.5	48.8	104.2	73.9	0.970	101.44	44.31	102.82	52.72	0.861
HDL	34	11.2	31.3	10.2	33.2	9.9	0.649	32.19	10.46	31.61	9.99	0.731
Creatinine	2.2	1	2.1	1.8	1.1	0.9	0.362	2.11	1.56	1.9	1.68	0.416
HB	12.8	1	12.7	1.8	12	1	0.589	12.73	1.55	12.56	1.67	0.355
urea	72.9	49	46.1	27.2	32.9	34.3	0.013*	55.13	37.57	43.73	28.47	0.042*

FBS, Fasting blood sugar; 2HPP, 2-hour postprandial; TG, triglycerides; LDL, low-density lipoprotein; HDL, High-density lipoprotein; HB, Hemoglobin. Note: Some data on controls are missing. Bold values indicate statistical significance.

**Table 6** presents the association analyses between MALAT1 SNPs (rs619586 and rs3200401) and clinical characteristics in T2DM patients, showing that most traits were not significantly different across genotypes. Notably, rs619586 demonstrated a significant association with hemoglobin levels (genotype p = 0.039; allele p = 0.049), indicating potential hematological effects. For rs3200401, no traits reached statistical significance, though HDL was borderline (genotype p = 0.062), suggesting no significant genotype-trait links. These results imply a limited influence of these MALAT1 variants on most measured parameters in T2DM.

**Table 7** details the associations between MALAT1 SNPs (rs619586 and rs3200401) and clinical characteristics in DN patients, with most traits showing no significant differences across genotypes. The key significant findings were for rs3200401, which was associated with FBS (genotype p = 0.099, allele p = 0.018) and 2HPP (genotype p = 0.034, allele p = 0.019), suggesting thatthis variant influences glycemic control in DN. For rs619586, all traits were non-significant, indicating no strong genotype-trait associations beyond the noted glycemic measures. These patterns highlight rs3200401’s potential relevance to glucose dysregulation, specifically in DN.

**Table 8** examines associations between ANRIL SNPs (rs10965215 and rs10738605) and clinical traits in T2DM patients, revealing mostly non-significant results across genotypes. Significant findings include rs10965215 with 2HPP (genotype p = 0.024) and borderline effects on FBS (p = 0.050) and HB (p = 0.051), as well as allele-level significance for HB (p = 0.018). rs10738605 showed significance for FBS (genotype p = 0.021) and borderline for cholesterol (p = 0.070), with no allele-level significance noted in the visible data. These findings suggest ANRIL variants primarily relate to glycemic and minor hematological/metabolic traits in T2DM.

**Table 9** analyzes associations between ANRIL SNPs (rs10965215 and rs10738605) and clinical traits in DN patients, with predominantly non-significant results across genotypes. The primary significant finding was for rs10738605 with urea (genotype p = 0.013; allele p = 0.042), suggesting a potential link to renal function. All other traits, including FBS, 2HPP, CHOL, TG, LDL, HDL, creatinine, HB, and DM duration, showed p > 0.05 for both SNPs. No other traits reached significance, emphasizing that ANRIL variants in DN mainly correlate with urea levels rather than broader glycemic or lipid profiles.

## 4 Discussion

Diabetic kidney disease is one of the serious complications of T2DM that leads to progressive kidney damage and worsening of kidney function. The exact molecular mechanism of DN remains unclear; however, cooperation between environmental and genetic factors may contribute to its pathogenesis (19). Recent findings have demonstrated MALAT1’s role in DN progression. For example, ***Li et al.*** found that MALAT1 levels were increased and that methyltransferase G9a was recruited to reduce klotho expression in high-glucose-stimulated glomerular endothelial cell injury (20). Furthermore, another study reported that the MALAT1/miR-15b-5p/TLR4 signaling Axis was closely associated with the development of DN (21). Furthermore, previous studies have shown that MALAT1 upregulation is associated with endothelial dysfunction and hyperglycemia-induced inflammation (22).

In particular, numerous studies havedemonstratedANRIL’s role in the progression of DN. ***Sooshtari et al.***reported that ANRIL could regulate multiple molecules associated with DN, underscoring its importance as a therapeutic target for DN (15). Furthermore, it has been confirmed that ANRIL helps pyroptosis and renal injury in DN (23).

The current study aims to investigate the association of MALAT1 rs619586 and rs3200401, and ANRIL rs10965215 and rs10738605, with susceptibility to T2DM and DN in an Egyptian population, and to examine whether these variants correlate with clinical and laboratory measures relevant to renal involvement.

This study demonstrates that the MALAT1 rs619586 GG genotype and G allele were associated with reduced T2DM and DN risk compared to controls, whereas MALAT1 rs3200401 CT/TT genotypes, ANRIL rs10965215 GA/AA, and rs10738605 CG genotypes increased disease susceptibility. Importantly, none of these SNPs was associated with DN progression among T2DM patients, suggesting these variants influence disease initiation rather than progression from diabetes to nephropathy.

Our findings align with prior studies showing that the MALAT1 rs619586 GG genotype and G allele confer protection against T2DM (12), coronary artery disease, and ischemic stroke (24). Notably, Fathy et al. confirmed this protective effect specifically in Egyptians, supporting the relevance of our results to this population (25).

Our results suggested that the MALAT1 rs3200401 CT or TT genotypes were associated with susceptibility to T2DM and DN, which agreed with previous studies that verified a significant association between rs3200401 C > T and various cancers such as risk of colorectal cancer (26), gastric cancer (27), esophageal squamous cell carcinoma (28)and non-small cell lung cancer (29). Interestingly, Fathy et al. proved that the CT + TT genotypes of rs3200401 were associated with cerebral ischemic stroke in the Egyptian population (25).

Prior experimental studies have shown that MALAT1 is upregulated in diabetic nephropathy and contributes to podocyte dysfunction, β-catenin activation, and disruption of slit diaphragm integrity (30). Other work has also linked MALAT1 to renal tubular injury through LIN28A/Nox4/AMPK/mTOR signaling, reinforcing its role as a mediator of diabetic renal damage (8). Therefore, the association of rs619586 with lower disease risk may reflect functional effects on MALAT1 expression or stability, although this should be confirmed in future expression-based studies.By contrast, the increased risk associated with rs3200401 aligns with earlier genetic studies in T2DM and with evidence that MALAT1 participates in inflammatory and fibrotic pathways relevant to diabetic complications (8, 30),. The same variant has also been implicated in other diseases, suggesting that it may alter lncRNA function in a context-dependent manner (26). In our study, rs3200401 was associated with both T2DM and DN, but not with progression from T2DM to DN, which suggests a stronger role in disease susceptibility than in nephropathy progression. This pattern is consistent with the idea that MALAT1-related genetic effects may act early in the development of metabolic disease rather than during later renal deterioration.

Regarding ANRIL SNPs, our results show a significant association between the AG or AA genotypes of rs10965215 and susceptibility to T2DM and DN. Furthermore, the CG genotype at rs10738605 was significantly associated with an increased risk of T2DM and DN. In addition, the progressively stronger LD between ANRIL SNPs in DN patients (D’ = 0.90) vs. controls (D’ = 0.125) suggests these markers may tag disease-associated haplotypes, as evidenced by the protective GG haplotype (OR = 0.315; **Table 5**). However, fine-mapping studies are required to identify potential causal variants within this locus.

To the best of our knowledge, the current study is the first to consider the associations between ANRIL SNPs (rs10965215 and rs10738605) and T2DM or DN susceptibility. Few studies have examined the relationship between the abovementioned SNPs and different diseases. Cheng et al. have indicated that the A allele of rs10965215 and the G allele of rs10738605 were associated with an increased risk of myocardial infarction. They speculated that these polymorphisms could influence ANRIL expression by altering its secondary structure and stability (31). Furthermore, another study verified that rs10965215 (but not rs10738605) was associated with ischemic stroke predisposition (25).

Experimental evidence suggests that ANRIL contributes to diabetic renal injury by regulating extracellular matrix accumulation, VEGF, apoptosis, and inflammatory pathways (15, 32). ANRIL knockout studies further support a pathogenic role, as ANRIL deletion reduced albuminuria, polyuria, and diabetes-induced transcriptomic changes in the kidney (15). Together, these data suggest that ANRIL polymorphisms may modify the risk of diabetic complications by influencing the regulatory functions of this lncRNA.

Interestingly, none of the four SNPs was associated with DN progression among patients with T2DM. These findings may indicate that these polymorphisms affect susceptibility to T2DM and DN independently, rather than determining the transition from diabetes to overt nephropathy. Another possible explanation is that progression to DN is driven more strongly by environmental factors, glycemic control, disease duration, and hemodynamic or inflammatory stress than by the studied SNPs alone. The modest sample size and single-center design may also have limited power to detect progression-related effects.

The observed associations between specific MALAT1 and ANRIL polymorphisms and clinical traits in T2DM and DN patients reveal little genetic influences on disease phenotypes. In T2DM, MALAT1 rs619586 was significantly associated with hemoglobin levels (p=0.045 for genotype, p=0.049 for allele). In contrast, ANRIL rs10965215 was associated with 2HPP (p=0.024) and rs10738605 with FBS (p=0.021), suggesting that these lncRNA variants may modulate glycemic control and erythropoiesis independently of broader metabolic disruption. In DN patients, MALAT1 rs3200401 showed stronger glycemic effects (FBS: p=0.099/0.018; 2HPP: p=0.034/0.019), and ANRIL rs10738605 was linked to urea (p=0.013/0.042), potentially implicating these SNPs in renal stress responses. These selective associations align with lncRNAs’ established roles in inflammation and endothelial dysfunction, though the lack of widespread significance warrants validation in larger cohorts with functional studies.

Several limitations should be acknowledged. First, some SNPs deviated from the Hardy–Weinberg equilibrium in the control group, which may reflect sample size limitations, population structure, or control selection issues and warrants replication in larger cohorts. Second, the study was conducted at a single center, which may limit generalizability. Third, expression levels of MALAT1 and ANRIL were not measured, and thus the functional consequences of the studied polymorphisms could not be directly assessed.

Overall, our findings strengthen the evidence that MALAT1 and ANRIL are involved in diabetes-related kidney injury and suggest that selected variants in these genes may serve as early genetic markers of susceptibility. Future studies should validate these associations in larger multiethnic cohorts and combine genotyping with lncRNA expression analyses to clarify the functional impact of these variants.

## 5 Conclusions

Our findings suggest that MALAT1 rs619586 may be protective, whereas MALAT1 rs3200401 and ANRIL rs10965215/rs10738605 may increase susceptibility to T2DM and DN in the studied Egyptian population. However, these polymorphisms were not associated with DN progression among patients with T2DM, indicating that their main relevance may lie in disease susceptibility rather than progression. Therefore, these variants may have potential value as early risk biomarkers, pending validation in larger, multi-center studies.

## Data Availability

The datasets presented in this study can be found in online repositories. The names of the repository/repositories and accession number(s) can be found in the article/**Supplementary Material**.

## References

[1] Wang J Pi H Sun Q . The relationship between serum lipid levels and diabetic nephropathy in patients with primary diabetes. BMC Nephrol. (2025) 26:608. doi: 10.1186/s12882-025-04546-w 41168724 PMC12577230

[2] McGrath K Edi R . Diabetic kidney disease: diagnosis, treatment, and prevention. Am Fam Phys. (2019) 99:751–9. 31194487

[3] Xie K Cao H Ling S Zhong J Chen H Chen P . Global, regional, and national burden of chronic kidney disease, 1990-2021: a systematic analysis for the global burden of disease study 2021. Front Endocrinol Lausanne. (2025) 16:1526482. doi: 10.3389/fendo.2025.1526482 40110544 PMC11919670

[4] Zhang L Jiang L Xu R Zhang X Zhang B Yue R . Epidemiological research on diabetic nephropathy at global, regional, and national levels from 1990 to 2021: an analysis derived from the global burden of disease 2021 study. Front Endocrinol Lausanne. (2025) 16:1647064. doi: 10.3389/fendo.2025.1647064 40937407 PMC12420290

[5] Ratan Y Rajput A Pareek A Pareek A Singh G . Comprehending the role of metabolic and hemodynamic factors alongside different signaling pathways in the pathogenesis of diabetic nephropathy. Int J Mol Sci. (2025) 26:3330. doi: 10.3390/ijms26073330 40244213 PMC11989741

[6] Guo Y Feng Y Wu H Gao H . The role of non-coding RNAs in the pathogenesis and progression of diabetic kidney disease. Int J Mol Sci. (2026) 27:2352. doi: 10.3390/ijms27052352 41828573 PMC12985135

[7] Huang H Zhang G Ge Z . lncRNA MALAT1 promotes renal fibrosis in diabetic nephropathy by targeting the miR-2355-3p/IL6ST axis. Front Pharmacol. (2021) 12:647650. doi: 10.3389/fphar.2021.647650 33995063 PMC8117091

[8] Song P Chen Y Liu Z Liu H Xiao L Sun L . LncRNA MALAT1 aggravates renal tubular injury via activating LIN28A and the Nox4/AMPK/mTOR signaling axis in diabetic nephropathy. Front Endocrinol Lausanne. (2022) 13:895360. doi: 10.3389/fendo.2022.895360 35813614 PMC9259889

[9] Abdel-Azeez HA Kamel LM Mounir AAM Alhai NAA Atef DM . Circulating long non-coding RNAs MALAT1 and CASC2 as biomarkers for diabetic nephropathy in type 2 diabetic patients. Egypt J Intern Med. (2025) 37:187. doi: 10.1186/s43162-025-00579-7 38164791

[10] Mahmoud AK Abd El Moety MA khalifa Ahmed A EL-Sawy SA . MALAT1 gene expression in diabetic patients with or without nephropathy. SVU-International J Med Sci. (2023) 6:256–65. doi: 10.21608/svuijm.2023.197120.1547

[11] Abdulle LE Hao JL Pant OP Liu XF Zhou DD Gao Y . MALAT1 as a diagnostic and therapeutic target in diabetes-related complications: a promising long-noncoding RNA. Int J Med Sci. (2019) 16:548–55. doi: 10.7150/ijms.30097 31171906 PMC6535662

[12] Chang WW Zhang L Wen LY Huang Q Tong X Tao YJ . Association of tag single nucleotide polymorphisms (SNPs) at lncRNA MALAT1 with type 2 diabetes mellitus susceptibility in the Chinese Han population: a case-control study. Gene. (2023) 851:147008. doi: 10.1016/j.gene.2022.147008 36283602

[13] Hamdy SM Ibrahim HA Abdelaleem OO Shaker OG Hussein SK Massoud SM . MALAT1 SNP (rs619586) shows a protective effect against type 1 diabetes mellitus, while the miR-146a SNP (rs57095329) is linked to an increased risk of developing the disease. Mol Biol Rep. (2025) 52:340. doi: 10.1007/s11033-025-10404-7 40138023

[14] Moglad E Kaur P Menon SV Abida Ali H Kaur M . ANRIL's epigenetic regulation and its implications for cardiovascular disorders. J Biochem Mol Toxicol. (2024) 38. doi: 10.1002/jbt.70076 39620406

[15] Sooshtari P Feng B Biswas S Levy M Lin H Su Z . ANRIL regulates multiple molecules of pathogenetic significance in diabetic nephropathy. PloS One. (2022) 17:e0270287. doi: 10.1371/journal.pone.0270287 35984863 PMC9390929

[16] Li F Ma Z Cai Y Zhou J Liu R . Optimizing diabetic kidney disease animal models: insights from a meta‐analytic approach. Anim Model Exp Med. (2023) 6:433–51. doi: 10.1002/ame2.12350 37723622 PMC10614131

[17] Bajaj M McCoy RG Balapattabi K Bannuru RR Bellini NJ Bennett AK . Summary of revisions: standards of care in diabetes—2026. Diabetes Care. (2026) 49:S6–S12. doi: 10.2337/dc26-SREV 41358896 PMC12690167

[18] Del Vecchio L Cases A Eisenga MF Małyszko J Barratt J Bolignano D . KDIGO 2026 clinical practice guideline for anemia in chronic kidney disease (CKD): a commentary from the European Renal Best Practice (ERBP). Nephrol Dial Transplant. (2026) 2026:gfag014. 10.1093/ndt/gfag014 PMC13423824 41604211

[19] Zhang L Long J Jiang W Shi Y He X Zhou Z . Trends in chronic kidney disease in China. N Engl J Med. (2016) 375:905–6. doi: 10.1056/NEJMc1602469 27579659

[20] Li Y Ren D Xu G . Long noncoding RNA MALAT1 mediates high glucose-induced glomerular endothelial cell injury by epigenetically inhibiting klotho via methyltransferase G9a. IUBMB Life. (2019) 71:873–81. doi: 10.1002/iub.2009 30762931

[21] Yang Z Song D Wang Y Tang L . lncRNA MALAT1 promotes diabetic nephropathy progression via miR-15b-5p/TLR4 signaling axis. J Immunol Res. (2022) 2022:8098001. doi: 10.1155/2022/8098001 35910856 PMC9334040

[22] Puthanveetil P Chen S Feng B Gautam A Chakrabarti S . Long non-coding RNA MALAT1 regulates hyperglycaemia induced inflammatory process in the endothelial cells. J Cell Mol Med. (2015) 19:1418–25. doi: 10.1111/jcmm.12576 25787249 PMC4459855

[23] Wang J Zhao S-M . LncRNA-antisense non-coding RNA in the INK4 locus promotes pyroptosis via miR-497/thioredoxin-interacting protein axis in diabetic nephropathy. Life Sci. (2021) 264:118728. doi: 10.1016/j.lfs.2020.118728 33160992

[24] Wang G Li Y Peng Y Tang J Li H . Association of polymorphisms in MALAT1 with risk of coronary atherosclerotic heart disease in a Chinese population. Lipids Health Dis. (2018) 17:75. doi: 10.1186/s12944-018-0728-2 29631611 PMC5891990

[25] Fathy N Kortam MA Shaker OG Sayed NH . Long noncoding RNAs MALAT1 and ANRIL gene variants and the risk of cerebral ischemic stroke: an association study. ACS Chem Neurosci. (2021) 12:1351–62. doi: 10.1021/acschemneuro.0c00822 33818067

[26] Li K Han Z Wu J Ye H Sun G Shi J . The relationship between MALAT1 polymorphism rs3200401 C > T and the risk of overall cancer: a meta-analysis. Med Kaunas. (2022) 58. doi: 10.3390/medicina58020176 35208500 PMC8879331

[27] Hong JH Jin E-H Chang IA Kang H Lee S-I Sung JK . Association of long noncoding RNA MALAT1 polymorphisms with gastric cancer risk in Korean individuals. Mol Genet Genom Med. (2020) 8:e1541. doi: 10.1002/mgg3.1541 33135867 PMC7767557

[28] Qu Y Shao N Yang W Wang J Cheng Y . Association of polymorphisms in MALAT1 with the risk of esophageal squamous cell carcinoma in a Chinese population. Onco Targets Ther. (2019) 12:2495–503. doi: 10.2147/OTT.S191155 31040692 PMC6452823

[29] Tong G Tong W He R Cui Z Li S Zhou B . MALAT1 polymorphisms and lung cancer susceptibility in a Chinese Northeast Han population. Int J Med Sci. (2022) 19:1300–6. doi: 10.7150/ijms.73026 35928715 PMC9346381

[30] Hu M Wang R Li X Fan M Lin J Zhen J . LncRNA MALAT1 is dysregulated in diabetic nephropathy and involved in high glucose-induced podocyte injury via its interplay with β-catenin. J Cell Mol Med. (2017) 21:2732–47. doi: 10.1111/jcmm.13189 28444861 PMC5661111

[31] Cheng J Cai MY Chen YN Li ZC Tang SS Yang XL . Variants in ANRIL gene correlated with its expression contribute to myocardial infarction risk. Oncotarget. (2017) 8:12607–19. doi: 10.18632/oncotarget.14721 28107200 PMC5355039

[32] Thomas AA Feng B Chakrabarti S . ANRIL regulates production of extracellular matrix proteins and vasoactive factors in diabetic complications. Am J Physiol Endocrinol Metab. (2018) 314:E191–200. doi: 10.1152/ajpendo.00268.2017 29118015

