# Supplementary material for: Associations of MALAT1 rs619586/rs3200401 and ANRIL rs10965215/rs10738605 polymorphisms with type 2 diabetes and diabetic nephropathy risk in an Egyptian population
Source: Front Endocrinol (Lausanne). 2026 Jun 12;17:1802490. doi: 10.3389/fendo.2026.1802490 (PMC13346653; doi:10.3389/fendo.2026.1802490)
Supplement: Supplementary file 1 [file DataSheet1.pdf]

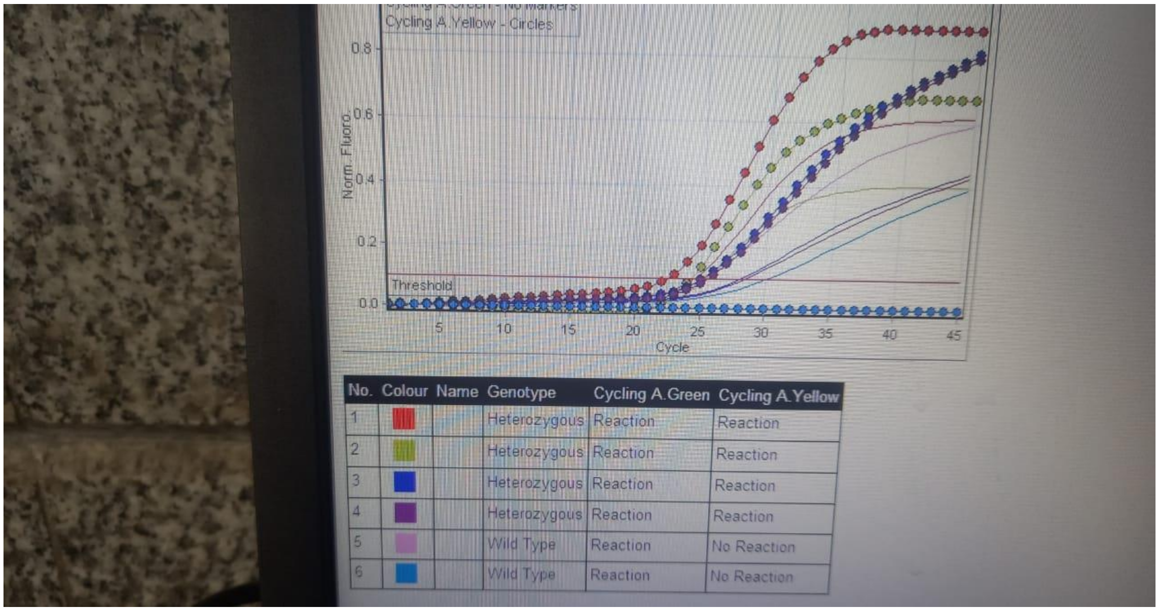
Cycling A.Yellow - Circles
Norm. Fluoro.
0.8
0.6
0.4
0.2
0.0
Threshold
5
10
15
20
25
30
35
40
45
Cycle
No. Colour Name Genotype Cycling A.Green Cycling A.Yellow
1 Heterozygous Reaction Reaction
2 Heterozygous Reaction Reaction
3 Heterozygous Reaction Reaction
4 Heterozygous Reaction Reaction
5 Wild Type Reaction No Reaction
6 Wild Type Reaction No Reaction

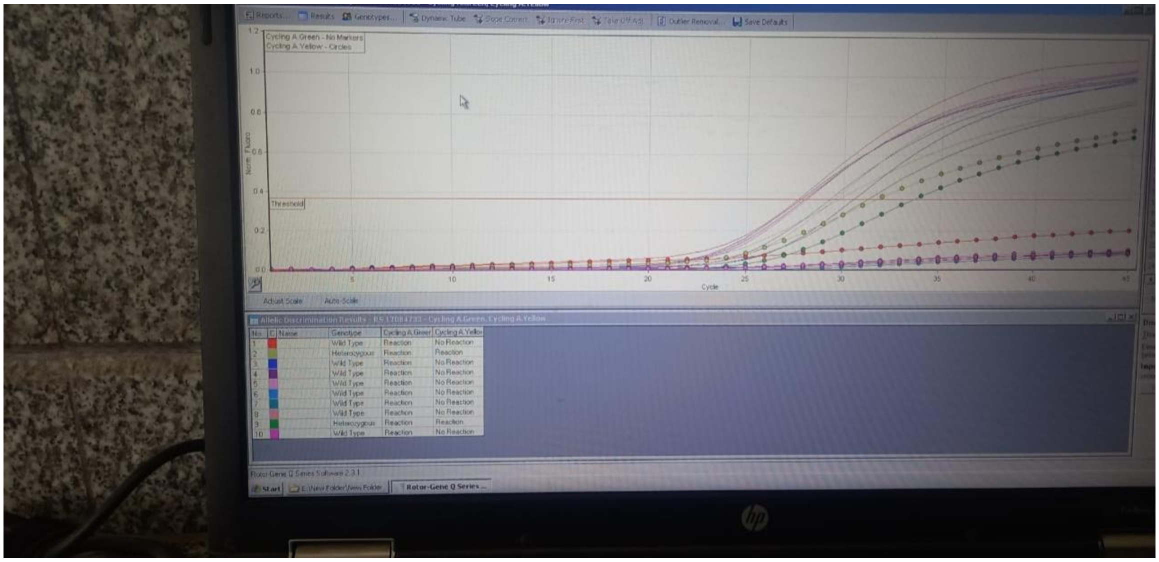
Cycling A.Green - No Markers
Cycling A.Yellow - Circles
Threshold
Adjust Scale
Auto-Scale
No. C Name Genotype Cycling A.Green Cycling A.Yellow
1 Wild Type Reaction No Reaction
2 Heterozygous Reaction Reaction
3 Wild Type Reaction No Reaction
4 Wild Type Reaction No Reaction
5 Wild Type Reaction No Reaction
6 Wild Type Reaction No Reaction
7 Wild Type Reaction No Reaction
8 Wild Type Reaction No Reaction
9 Heterozygous Reaction Reaction
10 Wild Type Reaction No Reaction
Rotor-Gene Q Series Software 2.3.1
Start
Rotor-Gene Q Series ...
hp

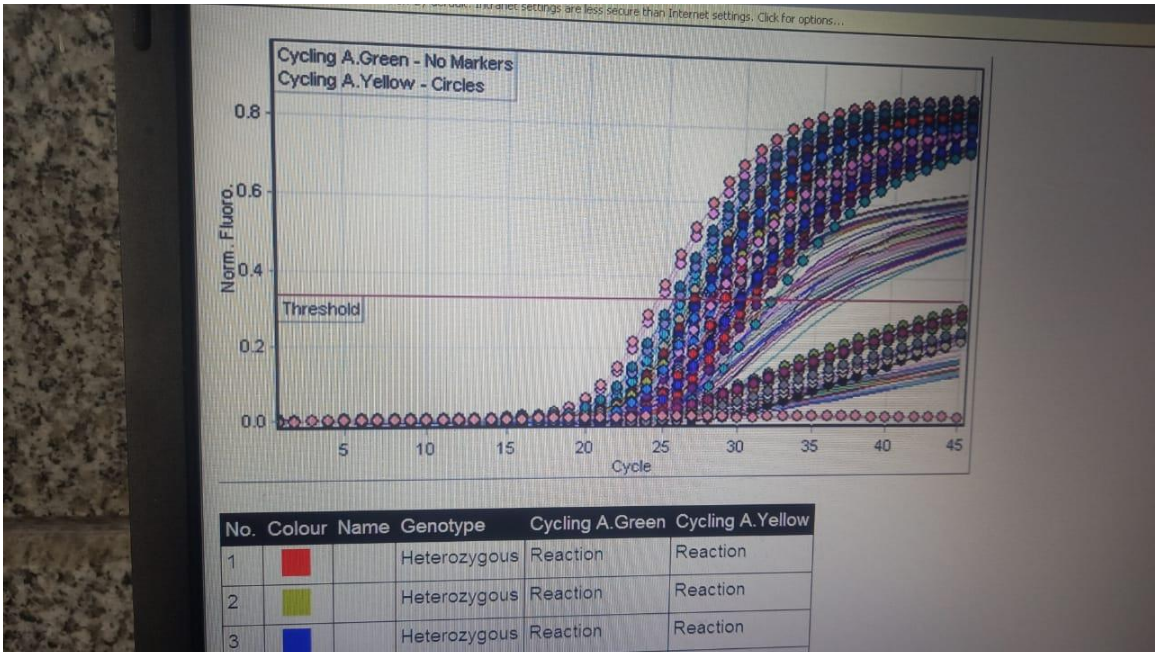

No.	Colour	Name	Genotype	Cycling A.Green	Cycling A.Yellow
1			Heterozygous	Reaction	Reaction
2			Heterozygous	Reaction	Reaction
3			Heterozygous	Reaction	Reaction
